# Task and information conflicts in the numerical Stroop task

**DOI:** 10.1111/psyp.14057

**Published:** 2022-03-30

**Authors:** Ronen Hershman, Lisa Beckmann, Avishai Henik

**Affiliations:** ^1^ Department of Cognitive and Brain Sciences Ben‐Gurion University of the Negev Beer‐Sheva Israel; ^2^ Zlotowski Center for Neuroscience Ben‐Gurion University of the Negev Beer‐Sheva Israel; ^3^ Department of Psychology University of Konstanz Konstanz Germany; ^4^ Department of Psychology Ben‐Gurion University of the Negev Beer‐Sheva Israel

**Keywords:** cognitive control, information conflict, numerical cognition, pupillometry, task conflict

## Abstract

Studies of the Stroop color‐word task have provided evidence for the existence of two conflicts: (1) an early task conflict between noting the relevant color and reading afforded by the irrelevant word (or word‐like stimuli), and (2) a late information conflict between the information provided by the word and the information provided by the color. Measurements of pupil changes, in addition to reaction time (RT), have extended understanding regarding these two conflicts. The current work examines the generalizability of such understanding. We ask whether similar processes work in the comparative judgment of numbers (e.g., in the numerical Stroop task). We present two experiments that support and extend the knowledge gained in the word‐color context to numerical processing. Similar to results with the Stroop color‐word task, we found a dissociation between RT and pupillometry and an early task conflict followed by an information conflict.

## Abstract 2

Recent Stroop color‐word studies have indicated the existence of an early task conflict followed by an information conflict. The current experiments used pupillometry to show the existence of these two conflicts in a numerical Stroop‐like task. Accordingly, our research extends and generalizes the two‐conflict notion beyond color‐word processing.

## 1 INTRODUCTION

### 1.1 Task and information conflicts in the numerical Stroop task

One of the popular cognitive control tasks is the Stroop color‐word task (Stroop, 1935). Participants are exposed to stimuli in color and asked to report the color of the stimulus and ignore its meaning. Commonly, there are three conditions: congruent (e.g., RED in red), neutral (e.g., XXXX or a color patch in red), and incongruent (e.g., BLUE in red). Incongruent trials lead to longer reaction time (RT) than neutral trials (i.e., interference), whereas congruent trials are either faster (facilitation) or similar in RT to neutral trials. Namely, the interference is large and robust and the facilitation is small and fragile (Hershman & Henik, 2019; Kalanthroff & Henik, 2013; MacLeod, 1991).

Throughout the years, the Stroop color‐word task became a paradigmatic control task and quite a few Stroop‐like tasks have been proposed and used. In the area of numerical cognition, Besner and Coltheart (1979) and Henik and Tzelgov (1982) studied the numerical Stroop task. In the numerical Stroop task, participants are exposed to two digits of different values and sizes and asked to decide which digit is larger than the other. Participants could be asked to pay attention to the physical size and ignore the numerical value of the digits or the other way around, pay attention to the numerical value and ignore the physical size. The numerical Stroop task is similar to the Stroop color‐word task because, in both tasks, participants are asked to pay attention to a physical dimension and ignore a symbolic dimension (i.e., words/letters and digits). Similar to the color‐word task, the physical task has three conditions; congruent (e.g., 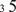, the physically smaller digit is smaller in value), neutral (e.g., 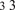, the digits have the same value but different sizes), and incongruent (e.g., 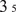, the physically larger digit is smaller in value). Also similar to the color‐word task, incongruent trials lead to longer RT than neutral trials (i.e., interference). However, unlike the color‐word task, congruent trials are commonly faster than neutral trials (i.e., facilitation). In this task, both interference and facilitation are large and robust.

Recent works on the Stroop color‐word task revealed the existence of two conflicts, the information conflict and the task conflict (Goldfarb & Henik, 2007; Kalanthroff et al., 2018; Monsell et al., 2001). Moreover, it has been suggested that the task conflict may be “responsible” for the seemingly fragile nature of the facilitation effect (Kalanthroff & Henik, 2013). Hence, we aimed to probe the generality of the existence of the task conflict by studying the physical version of the numerical Stroop task. We reasoned that the commonly found facilitation in the numerical Stroop task is due to the involvement of the task conflict.

### 1.2 The color‐word Stroop conflicts

It has been suggested that the interference in the color‐word Stroop task is due to two major conflicts. The information conflict appears due to contradicting information between word meaning and ink color in incongruent stimuli.^1^ No contradicting information exists in the congruent condition. In addition, stimuli evoke tasks that are strongly associated with them (Rogers & Monsell, 1995; Waszak et al., 2003). In particular, words tend to evoke reading (Monsell et al., 2001). Thus, the two tasks of word reading and naming the color compete. Such competition is triggered both in the incongruent and the congruent conditions. It is not triggered or evoked to a lesser degree by stimuli that are not words or word‐like. When using RT as a measure of performance, the information conflict has been shown repeatedly in many experiments (MacLeod, 1991). In contrast, the evidence for task conflict has been shown under limited conditions. Monsell et al. (2001) presented evidence that task set conflict appears when repetitions of irrelevant words are strictly controlled. Goldfarb and Henik (2007) reported that it was possible to expose the task conflict when control was dramatically reduced. Reduction of control was achieved by increasing the proportion of non‐word neutral trials (Tzelgov et al., 1992) and manipulating advanced information (i.e., priming) regarding the upcoming stimulation. Interestingly, a reduction of control could be found in the stop signal task, in trials in which participants responded to the target when they were supposed to withhold their response. When participants erroneously responded on stop trials, Kalanthroff et al. (2013) found evidence for task conflict facilitation. In quite a few studies (for review see Kalanthroff et al., 2018) task conflict was indicated by reverse facilitation. Namely, RT of congruent trials was longer than RT of neutral trials. Note that the reverse facilitation appears because congruent trials suffer from task conflict similarly to incongruent trials. In both conditions, participants need to play down reading in favor of noting the color. Goldfarb and Henik reasoned that task conflict needs to be controlled very early on in order to enable the performance of the task (i.e., responding to the color and not reading). Because task conflict is handled early in executing the goal task of reporting the color, task conflict has been very rarely exposed and is unnoticed in most experiments.

Reverse facilitation became a marker for task conflict (Kalanthroff et al., 2013, 2018; Kalanthroff & Henik, 2014; La Heij & Boelens, 2011). However, as suggested above, in order to show reverse facilitation, special experimental manipulations were required. In contrast, we recently found that it was possible to expose task conflict in a standard Stroop color‐word task by using pupillometry. We examined task and information conflicts using not only RT but also changes in pupil dilation. In a regular experimental design with equal presentation proportions of congruent, neutral and incongruent trials, pupil dilation presented evidence for task conflict with no special experimental manipulation (Goldfarb & Henik, 2007; Hershman & Henik, 2019, 2020; Kalanthroff et al., 2018).

### 1.3 Stroop conflicts and pupil dilation

Pupils increase due to exposure to dark stimuli (Ellis, 1981), however, pupil dilation is also considered to be an index of mental effort in cognitive control tasks in general (Kahneman & Beatty, 1966; van der Wel & van Steenbergen, 2018) and in the Stroop color‐word task in particular (Brown et al., 1999; Hershman & Henik, 2019; Laeng et al., 2011; Siegle et al., 2004). Temporal analysis of the pupil dilation shows that the indications for task conflict, measured by the difference between different neutral and congruent conditions, appear relatively early, whereas the information conflict, indicated by the divergence of the curves of the incongruent and congruent conditions, starts later (Hershman & Henik, 2019, 2020; Hershman et al., 2020, 2021). In particular, after about 500 ms post‐stimulus onset, the pupils were larger for congruent than for neutral trials (i.e., reverse facilitation and task conflict), and about 1000 ms post‐stimulus onset, the pupils were larger for incongruent than for congruent trials (the information conflict). Importantly, this indication for task conflict (i.e., the reverse facilitation) was eliminated when neutral trials composed of series of letters were replaced by trials with non‐color words like *lion* (Hershman et al., 2020; Hershman & Henik, 2019).

Unraveling the existence of task sets (Monsell et al., 2001) or task conflict (Goldfarb & Henik, 2007) led to discussions of what produces task conflict (Braverman et al., 2014; Hershman & Henik, 2019; Kalanthroff et al., 2018; La Heij & Boelens, 2011; Littman et al., 2019) and what enables researchers to find evidence for its existence. It is clear that the choice of neutral trials is derived from the conceptual basis or the framing of the Stroop color‐word task. For example, if one focuses on congruency (with no importance for neutral trials) and defines the Stroop effect as the RT difference between congruent and incongruent trials, then information conflict and nothing else is central to one's conceptualization of the task and the potential of interference. Recently, Hershman et al. (2021) suggested that meaningful or word‐like stimuli activate different degrees of task conflict. The more word‐like a stimulus is, the larger the potential for task conflict. We also suggested that color patches (the same stimuli used by Stroop in his original study) do not afford reading and accordingly, do not produce task conflict. Moreover, the use of pupillometry helps to reveal the task conflict, which might be hidden in RT experiments. Tracking the temporal changes of the pupil provides accurate information about Stroop conflicts in general and about the temporal occurrences of these conflicts in particular (Hershman et al., 2022). This leads to the question, what are the implications for the numerical Stroop task?

### 1.4 The numerical Stroop task and task conflict

Despite the considerable number of studies that deal with the conflicts of the Stroop color‐word task (i.e., both task and information conflicts), the reports of task conflict in the numerical Stroop task are limited (Ben‐Shalom et al., 2012). However, several pieces of evidence suggest the existence of early effects of irrelevant numerical processing on physical judgments. One such piece of evidence is the fact that irrelevant numerical information affects physical judgments even though in many cases physical judgments are faster than numerical judgments (Gebuis et al., 2009; Henik & Tzelgov, 1982; Rubinsten et al., 2002). In a functional magnetic resonance imaging (fMRI) and event‐related potentials (ERPs) study, Cohen Kadosh et al. (2007) found indications for an effect of the irrelevant numerical value 300 ms post‐stimulus onset. Szucs et al. (2009) conducted an ERP study and found evidence for congruency effects on the amplitude of ERPs as early as 180–210 ms and 270–300 ms post‐stimulus onset (fig. 8, p. 1970, in their study). A recent ERP study by Huang et al. (2021) reported a significant congruency effect on the N200 component that was composed of both facilitation (less negative N200) and interference. Moreover, in a recent study, Reike and Schwarz (2017) reported the effects of numerical values on the sensitivity to differences in physical sizes. They used a single‐digit comparative judgment of physical sizes of digits and applied a signal detection analysis to performance. Their results showed that physically small digits, presented in a set of physically small digits (i.e., the same digit in a slightly smaller vs. in a slightly larger size), were discriminated with higher sensitivity when they were numerically small than when they were numerically large. In contrast, physically large digits, presented in a set of physically large digits (i.e., the same digit in a large size vs. in a slightly larger size), were discriminated with higher sensitivity when they were numerically large than when they were numerically small. This suggested that the irrelevant numerical values produced changes in sensitivity in the physical judgment task.

In their original study, Henik and Tzelgov (1982) used the same digit in different physical sizes as the neutral condition of the physical comparison task (e.g., 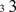 ). This made sense because similar to the congruent and incongruent conditions, this neutral condition was composed of digits, but different from the congruent and incongruent conditions, it did not have the interfering numerical difference between the two digits. Quite a few studies that followed this experimental design used similar neutral conditions (e.g., Cohen Kadosh et al., 2007; Szucs et al., 2009). A stimulus composed of the same digits in different physical sizes (e.g., 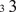 ) seems to be similar, in essence, to a word neutral (e.g., lion) in the color‐word task. However, our Stroop color‐word studies suggest that these conditions induce task conflict because of the presentation of the digits (or the words). In contrast, color patches would produce no task conflict in the context of the numerical Stroop task, similar to the color‐word context (Hershman et al., 2021).

Accordingly, we conducted two physical comparative judgment experiments. Participants were presented with congruent and incongruent numerical Stroop stimuli. The congruent and incongruent trials were the same in both experiments. The neutral conditions differed; colored patches (i.e., rectangles) of different physical sizes were used in experiment 1, and digits (similar to Henik & Tzelgov, 1982) were used in experiment 2. We used both RT and pupil dilation as dependent measures. In experiment 1, we expected pupil measurement to show early reverse facilitation (i.e., task conflict) followed by a late difference between incongruent and congruent trials (i.e., information conflict). RT was expected to show information conflict with no facilitation. In experiment 2, we expected pupil measurement to show information conflict, but no reverse facilitation (i.e., no task conflict). In contrast, RT was expected to show both information conflict and facilitation.

## 2 EXPERIMENT 1

Participants carried out the physical version of the numerical Stroop task (Henik & Tzelgov, 1982), with the ratio between the two digits being 0.5 (1 vs. 2, 2 vs. 4, and 4 vs. 8) for both the irrelevant numerical values and for the relevant physical sizes. These ratios were selected to avoid any difference between the processing difficulties of the two dimensions. Unlike the original task, the neutral stimuli were filled rectangles with a fixed width but different heights. Rectangles do not activate numerical processing, whereas digits do, accordingly, we expected a difference in pupil dilation between the neutral and congruent conditions (i.e., reverse facilitation). This difference would indicate the existence of task conflict.

### 2.1 Method

#### 2.1.1 Participants

Twenty‐one participants (13 females, mean age 23.72 years, *SD* = 1.15) from Ben‐Gurion University of the Negev participated in the experiment in return for 25 shekels (approximately $7) or course credit. The study was approved by the ethics committee of the Psychology Department. All participants signed an informed consent form prior to participation in the experiment. Each participant had normal vision (those with glasses or contact lenses were excluded) as well as normal color vision and no reported history of attention deficit disorder or any learning disabilities.

#### 2.1.2 Stimuli

Participants were presented with two red‐colored single‐digit numbers (Arial font, RGB: 255, 0, 0) that could be congruent (i.e., the numerically smaller digit was physically smaller) or incongruent (i.e., the numerically smaller digit was physically larger). The numerical ratio between the two digits that appeared was always 0.5 (specifically, the digits used were: 1 & 2, 2 & 4, and 4 & 8). The physical size distance of the digits was also a ratio of 0.5 (e.g., 1 always appeared half as large as 2 in the congruent condition or twice as large as 2 in the incongruent condition). For the neutral condition, participants were presented with two filled rectangles having different heights, but the same fixed width of 100 pixels. The ratio between the heights was always 0.5 (equivalent to the numerical stimuli of 1 & 2, 2 & 4, and 4 & 8), and was represented by multiplying the physical value by 80 pixels (e.g., the physical value of 2 would be represented by [2 × 80 =] 160 pixels in height).

The conditions and the stimuli within each condition were selected randomly but were balanced. The presented stimuli (see Table 1) appeared against a silver (RGB: 192, 192, 192) background. Note that perceptual features of the colored rectangles were selected in order to trigger less pupil constriction compared to other stimuli. Specifically, the area of these stimuli was larger than the size of the digits and as a result, these stimuli were darker than the background. In total, the brightness of the rectangles was less than that of the other stimuli, and less brightness (or more darkness) leads to larger pupil dilation (Ellis, 1981). In addition, the spatial frequency of the colored rectangles was smaller than that of the other stimuli and low spatial frequency (compared to high spatial frequency) leads to larger dilation of the pupil (Barbur & Thomson, 1987). Hence, if colored rectangles resulted in smaller dilation (compared to other conditions), it would not be due to their perceptual features.

**TABLE 1:** The presented stimuli in the experiments

Congruent and incongruent pairs	Neutral rectangles (Experiment 1)	Neutral digits (Experiment 2)
1 vs. 2	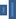 (1 vs. 2)	2 vs. 2
2 vs. 4	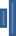 (2 vs. 4)	4 vs. 4
4 vs. 8	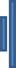 (4 vs. 8)	8 vs. 8

*Note*: The physical values represent multiplication with 80 and 40 pixels for experiments 1 and 2, respectively (e.g., here the physical value 2 represents [2 × 80 =] 160 pixels for the height of the value in experiments 1 and [2 × 40 =] 80 pixels in experiment 2). In the neutral condition of experiment 1, the values represent the height of the presented filled rectangles (with the width fixed at 100 pixels; symbolic representation in the table).

#### 2.1.3 Procedure

**FIGURE 1:**
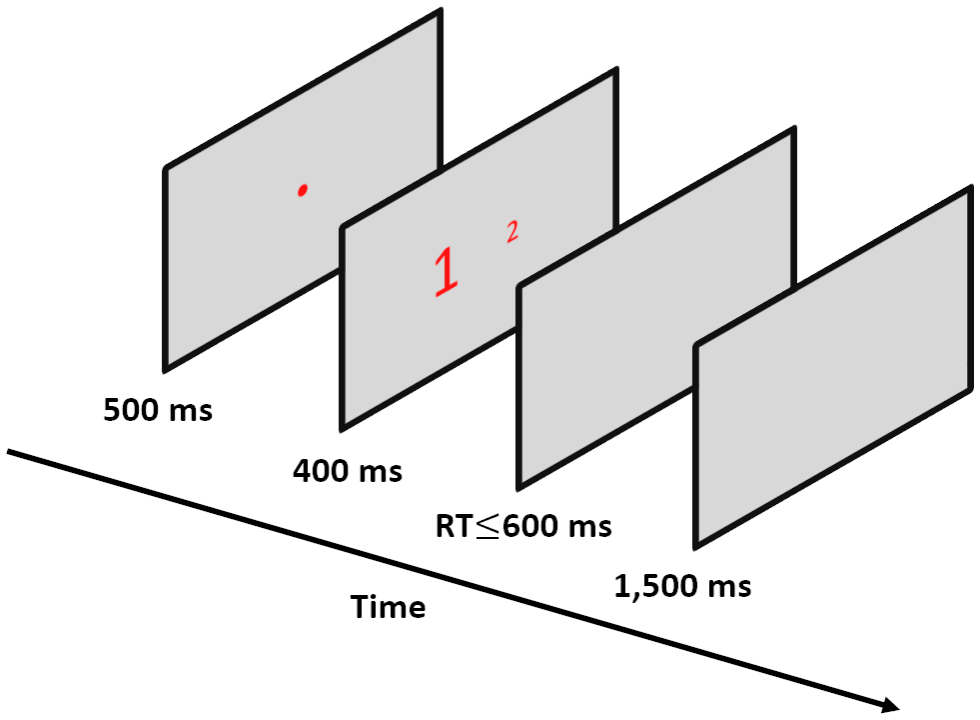
Example of a trial. Participants had to respond to the physically larger number and ignore the numerical dimension

The experiment was conducted in a dimly illuminated room and the participants were tested individually. A keyboard for measuring responses was placed in between the participant and a monitor on a table. Participants positioned their heads on a chinrest support to prevent head movement. The experiment included 10 practice trials that were excluded from the analysis. After each practice trial, participants received feedback on their accuracy. Participants had to have 80% correct trials in the practice to proceed to the experimental part. In the experimental part, participants carried out four blocks of 108 experimental trials each. At the beginning of each trial (see Figure 1 for a visual example) there was a 500 ms fixation in the form of a red circle in the center of the screen. The visual stimulus stayed in view for 400 ms and was followed by a blank screen for a maximum of 600 ms or until a keypress. Each trial ended with a 1500 ms inter‐trial‐interval (ITI) of a blank (silver) screen. Participants were asked to hit the “M” key with their right index finger if the stimulus on the right was physically larger, and to hit the “B” key with their left index finger if the stimulus on the left was physically larger. RT was calculated from the appearance of the visual stimulus to the reaction in the form of a keypress. Due to the length of the experiment, the participants had three breaks in which they were told that they could close their eyes. We asked them to keep their head static, resting on a chinrest.

#### 2.1.4 Apparatus

Pupil size was measured using a video‐based desktop‐mounted eye tracker (The Eye Tribe) with a sampling rate of 60 Hz (16.66 ms inter‐sampling time). Stimulus presentation and data acquisition were controlled by Psychtoolbox software (version 3.0.14) on MATLAB (MathWorks version 9.4.0.813654 [R2018a]). Stimuli were displayed on a 23‐inch LED monitor (Dell E2314Hf) at a resolution of 1920 × 1080 pixels, with a refresh rate of 60 Hz. The participant's head was positioned on a chin rest and the distance from the eyes to the monitor was set at about 50 cm. To maintain an accurate measurement of pupil size during the task, participants were required to keep their eyes fixated on the center of the screen and to avoid eye movements for the entire task. Pupil area was determined using the Eye Tribe algorithm.

#### 2.1.5 Pre‐processing of the pupillometry data

Two participants were excluded from the analysis because they did not have a minimum of 80 valid trials (correct responses with no more than 30% of missing values) in each condition. For the 19 remaining participants (12 females, mean age 24.63 years, *SD* = 2.31) included in the analysis, pupil data was processed using CHAP software (Hershman et al., 2019). First, pupil data was extracted from the Eye Tribe (pupil size in arbitrary units). Then, we removed outlier samples with *Z* scores larger than 2.5 (by using *Z* scores based on the mean and standard deviation calculated for each trial). Next, for each participant, we excluded from the analysis the trials with more than 30% of missing values. We also excluded trials with no response or with incorrect responses. This pre‐processing eliminated an average of 7.26% of trials. Next, we detected eye‐blinks by using Hershman et al.'s (2018) algorithm and filled missing values by using linear interpolation (Hershman & Henik, 2019). Next, time courses were aligned with the onset of the stimulus and divided by the baseline (baseline was defined as the average pupil size 500 ms before the stimulus onset).

### 2.2 Results

#### 2.2.1 Reaction time

**FIGURE 2:**
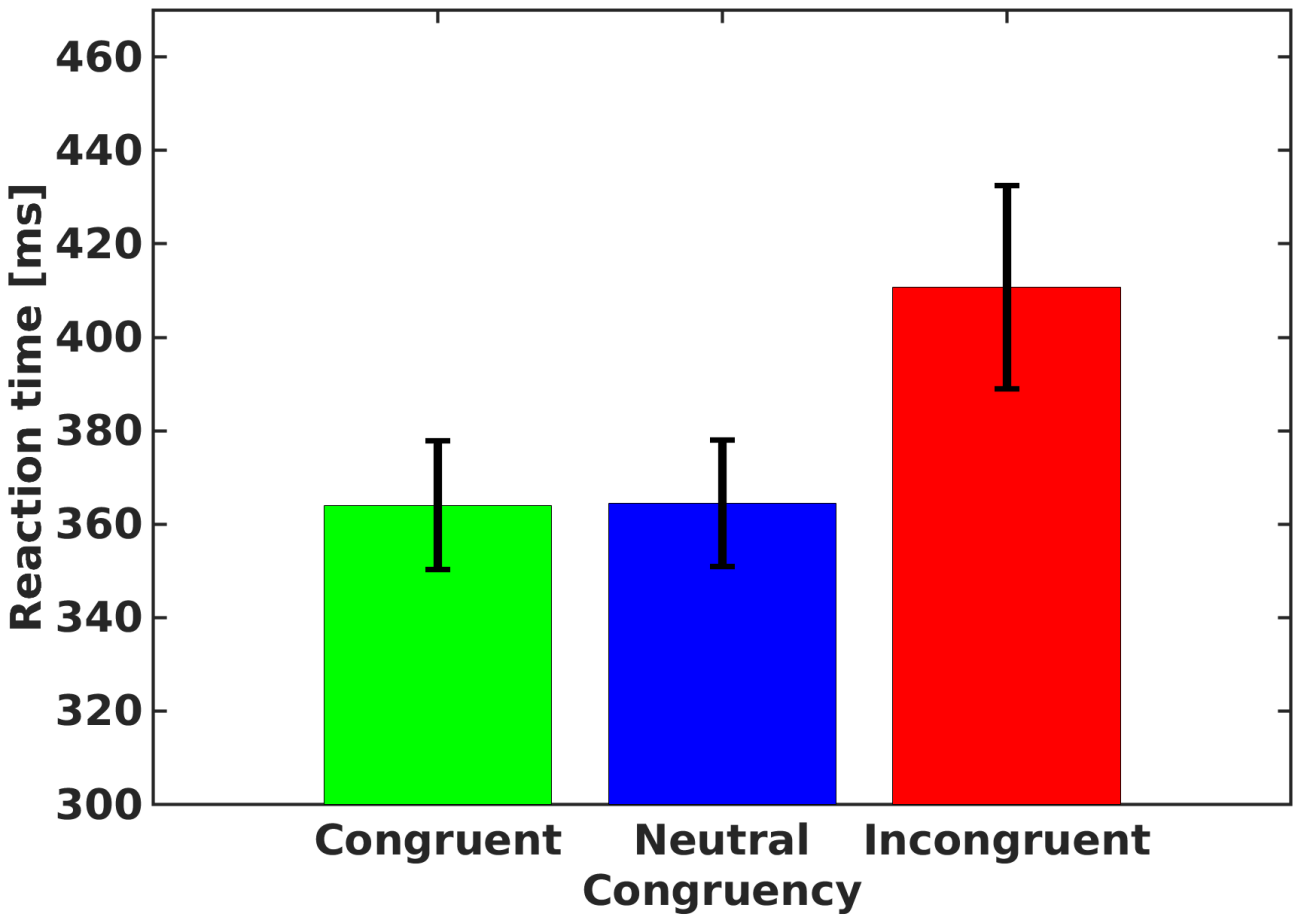
Mean reaction time for each congruency condition in experiment 1. Error bars represent 1 confidence interval from the mean

In order to verify that the task worked as expected, mean RTs of correct (and pupil valid) trials for each participant in each condition were subjected to a one‐way repeated measures analysis of variance (ANOVA) with congruency (congruent, incongruent, and neutral) as an independent factor (mean RTs in the various conditions are presented in Figure 2). As expected, an omnibus analysis produced a significant effect for congruency, $F\;\left(2,36\right)=47.06,p<.001,\eta_{p}^{2}=.72,{\mathrm{BF}}_{10}>1000$. Specifically, mean RT in incongruent trials was slower than in neutral trials, $F\;\left(1,18\right)=2,301,p<.001,{\mathrm{BF}}_{10}>1000,\text{Cohen}s^{\prime }d=1.919$. However, no differences were found between neutral and congruent trials, *F* (1, 18) < 1, ${\mathrm{BF}}_{10}=0.24,\;\text{Cohen}s^{\prime }d=0.017$. In other words, the RT analysis indicated the existence of information conflict, but no task conflict.

#### 2.2.2 Pupil size

**FIGURE 3:**
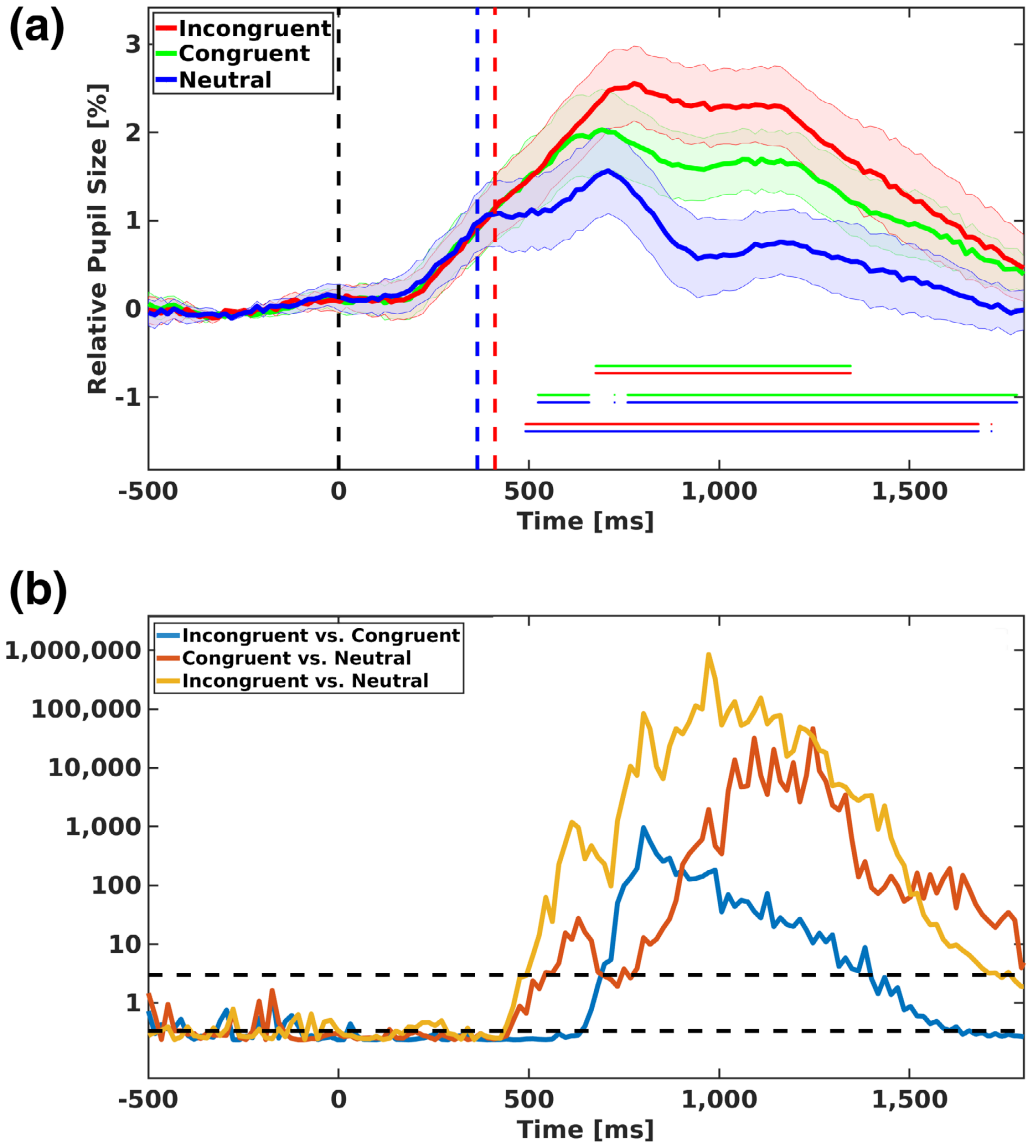
Mean relative pupil size and Bayes factors in experiment 1. (a) Mean relative pupil size (compared with pupil size at the stimuli onset) for the three congruency conditions of experiment 1 (participants had to indicate the physically larger digit). 0 represents stimuli onset and the vertical lines represent mean response times (around 400 ms post‐stimulus onset) for each condition. The three‐line curves present changes in pupil dilation as a function of time. The shaded areas represent 1 standard error from the mean. The horizontal lines represent meaningful differences (i.e., ${\mathrm{BF}}_{10}\geq 3$) for each contrast (e.g., the red and green lines indicate meaningful differences in pupil response between the incongruent and the congruent conditions). (b) Bayes factors (*BF*s) as a function of time for comparison between each two conditions in experiment 1. Each curve represents ${\mathrm{BF}}_{10}$ (namely, evidence for the alternative hypothesis that the two conditions are not the same). The horizontal black lines on 3 and 0.3 represent the threshold for decision making (${\mathrm{BF}}_{10}$ values above 3 provide evidence for the alternative hypothesis and ${\mathrm{BF}}_{10}$ values below 1/3 provide evidence for the null hypothesis). Please note, the scale for the *Y* axis is logarithmic

Mean relative changes of pupil size in each condition are presented in Figure 3a. In order to examine the temporal differences among the three conditions, we used Hershman and Henik's (2019) approach. Specifically, we compared each of the two conditions over the whole time‐course of pupil measurement. Meaningful differences (${\mathrm{BF}}_{10}\geq 3\;$) are presented in Figure 3a by the horizontal lines (e.g., the bottom double horizontal line presents meaningful differences between incongruent [red line] and neutral [blue line] conditions).

The analysis (Figure 3) indicates that pupil size was larger in incongruent trials compared with congruent trials. The differences between incongruent and congruent trials appeared at about 670 ms after the stimulus onset and were maintained for about 680 ms. In addition, pupil size was larger in both incongruent and congruent trials compared with neutral trials. These differences (with the neutral) were observed before the observation of the differences between incongruent and congruent trials. Specifically, the difference between incongruent and neutral trials appeared at about 490 ms after the stimulus onset and was maintained for about 1200 ms, and the difference between congruent and neutral conditions appeared at about 520 ms after the stimulus onset and was maintained until the end of the trial (between 660 and 760 ms post‐stimulus onset; BF was below 3 but still tended to support differences between the conditions—namely, BF was above 2).

#### 2.2.3 Analysis of conflict onset

To verify that the task conflict appeared before the information conflict, we carried out an analysis of conflict onset using smoothing data (made by using bins of ~67 ms). Specifically, for each participant, the onset of the task and of the information conflict were calculated separately. The onset of the task conflict was defined as the first divergence between congruent and neutral trials and the onset of the information conflict was defined as the first divergence between incongruent and neutral trials.^2^ Mean calculated onsets were subjected to a one‐tailed permutation t‐test with 10,000 permutations. Our analysis produced a significant effect, *p* = .02. In other words, our analysis suggested that the task conflict appeared before the information conflict.

### 2.3 Discussion

RTs were slower in incongruent trials compared to neutral trials, which were similar to congruent trials. In contrast, pupil dilation produced a different pattern. Specifically, pupil dilation presented both information (i.e., larger dilation in incongruent trials compared with congruent trials) and task (i.e., larger dilation in congruent trials than neutral trials) conflicts. Importantly, evidence for task conflict (i.e., reverse facilitation) appeared early on at about 520 ms after the stimulus onset. Moreover, the indication for task conflict appeared before the initiation of a congruency effect (i.e., a difference between congruent and incongruent trials). The latter, which is evidence for information conflict, appeared relatively late (about 700 ms after the stimulus onset). These results suggest that task conflict appears before the information conflict, similar to results found in pupillometry studies of the color‐word Stroop task (Hershman et al., 2020, 2021; Hershman & Henik, 2019, 2020).

One could argue that the difference between trials involving numbers and patch trials is just the difference in mental effort required to process each stimulus type. Moreover, numbers are processed at form, phonological, and semantic levels, and there are two numbers to compare that sometimes have different values (so the system may be more alert when numbers appear), but rectangles are less complex objects (at least in the context of the experiment where magnitude judgments are required) and the two different rectangles never have different semantic values. However, over all conditions of the experiment, no processing is required. Any kind of processing of stimuli besides their physical height is irrelevant to the task. Actually, for participants, it is not supposed to matter whether the stimuli are numbers or any other shapes. This is because it is irrelevant to the task that they are asked to complete—respond to the physical dimension of the stimuli. If less effort is required in the irrelevant task, it means that the irrelevant task is less dominant and therefore less task conflict is observed. In the present study, we choose colored rectangle stimuli, based on findings from the color‐word Stroop task in Hershman et al.'s (2021) study. In that study, we used colored patches (as in the present study) as well as abstract draws. Our findings showed that both meaningless types of neutral triggered less task conflict compared to non‐word letter strings (e.g., XXXX) that have morphological/phonological/orthographical meaning. Moreover, we found that colored patches triggered less task conflict compared to other neutral conditions (both letter strings and abstract draws). In other words, the more meaningless the stimulus is the less task conflict that will appear. Therefore, we decided to use rectangles (which have no meaning) instead of any other meaningful stimuli to increase the manipulation.

Another possible explanation for the present results is different arousal levels of the stimuli (Bradley et al., 2008, 2017; Partala & Surakka, 2003). However, again, this explanation is less likely because, across all the investigated conditions, the meaning of the stimuli was task‐irrelevant. Hence, if the meaning of the stimuli was processed, it would give us more evidence for the existence of task conflict because any kind of meaning of the stimuli, except their color, should not be processed.

The present findings, which echo those from the color‐word version of the Stroop task, suggest that numerical processing of symbolic numbers is an automatic process. Similar to the tendency to read upon exposure to words, there is a tendency to extract the meaning of numerical symbols upon exposure to such symbols. This tendency to extract meaning is automatic and is afforded by the stimuli. In contrast, the conflict between the information provided by the physical size and the numerical value appears later. Apparently, there is a need to accumulate information regarding the physical size and the (irrelevant) numerical value in order to create a conflict between them. What would happen if the neutral condition was composed of digits rather than a filled rectangle? As mentioned in the introduction, digits with the same numerical value are commonly used as neutrals in studies of the numerical Stroop task (Ashkenazi et al., 2009; Cohen Kadosh, 2008; Henik & Tzelgov, 1982). However, digits trigger numerical processing and as a result, they may induce task conflict. Namely, the use of neutral trials that contain digits (e.g., 2 & 2) would create task conflict similar to the congruent condition (e.g., 2 & 4) and thus would disable revealing the task conflict. Similar situations occur in the Stroop color‐word task when one uses a word as a neutral stimulus (Hershman et al., 2020, 2021; Hershman & Henik, 2019, 2020). To examine this possibility, we carried out an experiment with digits as neutrals. In this experiment, we expected to find no evidence for a task conflict.

## 3 EXPERIMENT 2

In this experiment, the neutral stimuli were composed of the same digit in two different physical sizes. These two‐digit neutrals replaced the rectangles in the previous experiment. In contrast with the rectangles, digits evoke numerical processing and thus trigger task conflict. However, the task conflict is expected both in the neutral and the congruent conditions. Hence, pupil dilation is not expected to show a difference between these conditions (no evidence for task conflict). Similar to quite a few reports in the past, RT should show facilitation.

### 3.1 Method

#### 3.1.1 Participants

Participation criteria were the same as in experiment 1 but this time we had 21 participants (13 females, mean age 23.72 years, *SD* = 1.15). The participants in experiment 2 were screened to verify they had not participated in experiment 1.

#### 3.1.2 Stimuli

The stimuli were identical to those used in experiment 1 with one important difference. Instead of filled rectangles, the neutral stimuli were digits with the same numerical value (i.e., 2 2, 4 4, and 8 8) but different physical sizes, created by multiplying one of the values in the pair by 40 pixels to create the on‐screen size (e.g., the physical value 2 would be represented by [2 × 40 =] 80 pixels).

#### 3.1.3 Procedure and apparatus

The procedure and apparatus were identical to those used in experiment 1.

#### 3.1.4 Pre‐processing of the pupillometry data

Data analysis was the same as in experiment 1. This resulted in the exclusion of two participants (because they did not have a minimum of 80 valid trials for each condition). For the 19 remaining participants (12 females, mean age = 23.74 years, *SD* = 1.19) included in the analysis, pre‐processing of pupil data eliminated an average of 8.17% of trials.

### 3.2 Results

#### 3.2.1 Reaction time

**FIGURE 4:**
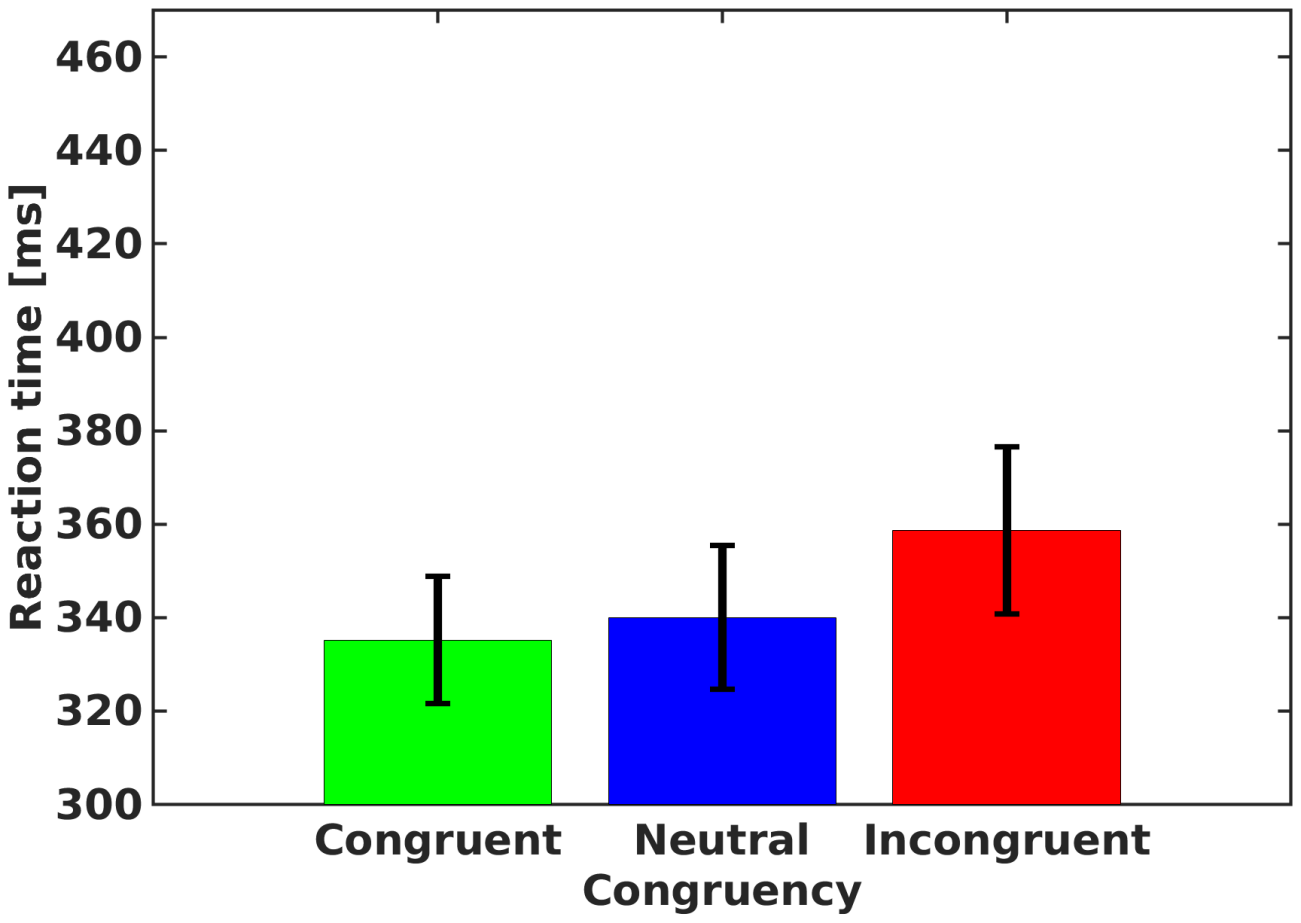
Mean reaction time for each congruency condition of Stroop trials in experiment 2. Error bars represent 1 confidence interval from the mean

Mean RTs of correct (pupil valid) trials for each participant in each condition were subjected to a one‐way repeated measures ANOVA with congruency (congruent, incongruent, and neutral) as an independent factor (mean RTs in the various conditions are presented in Figure 4). As expected, an omnibus analysis produced a significant effect of congruency, $F\;\left(2,36\right)=49.56,p<.001,\eta_{p}^{2}=.734,{\mathrm{BF}}_{10}>1000$. Specifically, mean RT was faster in congruent trials compared with neutral trials, $F\;\left(1,38\right)=5.39,p=.02,{\mathrm{BF}}_{10}=8.97,\text{Cohen}s^{\prime }\;d=0.446$, which were faster than incongruent trials, $F\;\left(1,38\right)=37.73,p<.001,{\mathrm{BF}}_{10}>1000,\text{Cohen}s^{\prime }\;d=1.717$.

#### 3.2.2 Pupil size

**FIGURE 5:**
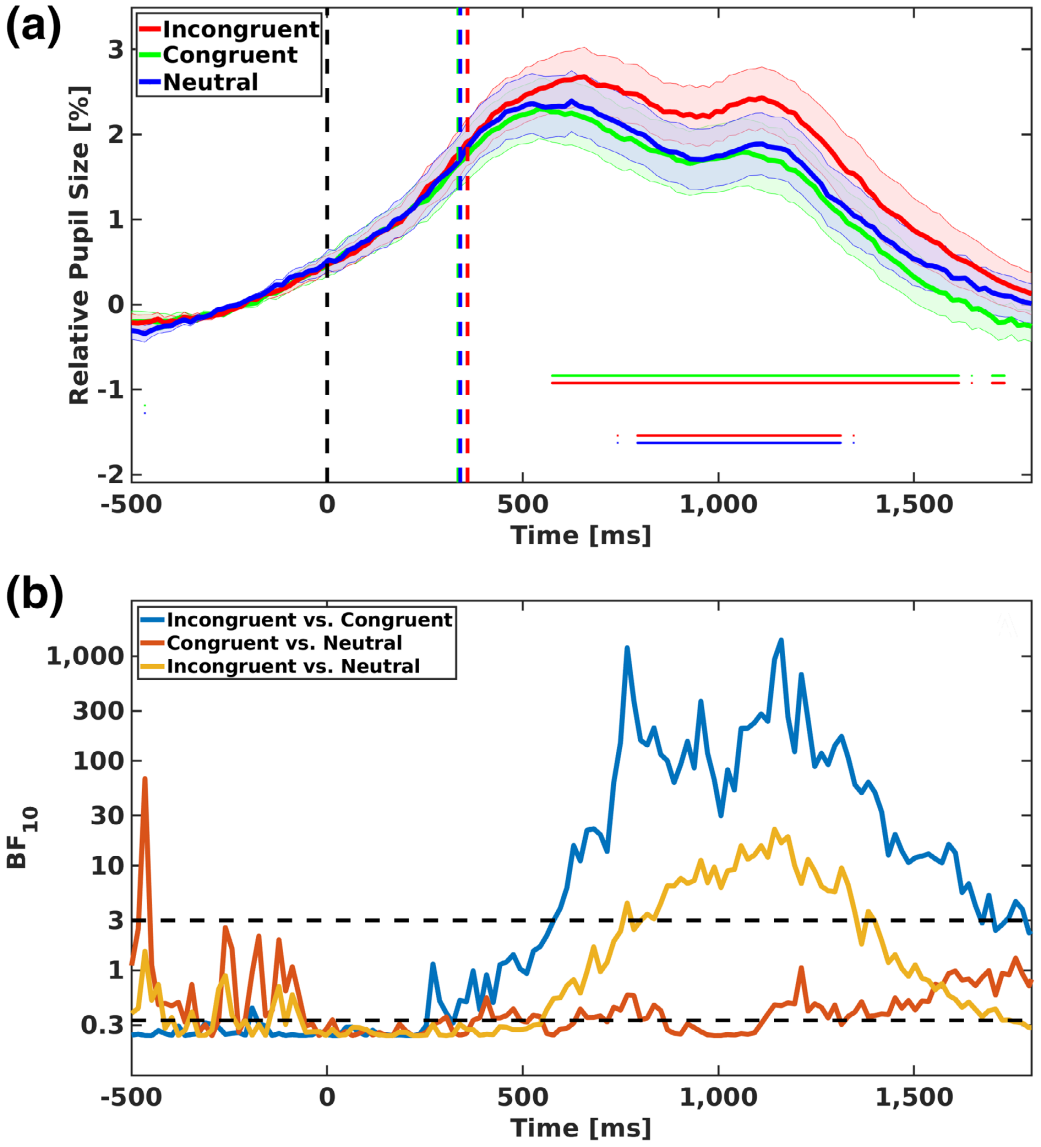
Mean relative pupil size and Bayes factors in experiment 2. (a) Mean relative pupil size (compared with pupil size at the stimuli onset) for the three congruency conditions of experiment 2. Participants had to indicate the larger digit in the physical dimension. 0 represents stimuli onset and the vertical lines represent mean response times (around 350 ms post‐stimulus onset) for each condition. The three‐line curves present changes in pupil dilation as a function of time. The shaded areas represent 1 standard error from the mean. The horizontal lines represent meaningful differences (i.e., ${\mathrm{BF}}_{10}\geq 3$) for each contrast (e.g., the red and green lines indicate meaningful differences in pupil response between the incongruent and the congruent conditions). (b) Bayes factors (*BF*s) as a function of time for comparison between each two conditions in experiment 2. Each curve represents ${\mathrm{BF}}_{10}$ (namely, evidence for the alternative hypothesis that the two conditions are not the same). The horizontal black lines on 3 and 0.3 represent the threshold for decision making (${\mathrm{BF}}_{10}$ values above 3 provide evidence for the alternative hypothesis and ${\mathrm{BF}}_{10}$ values below 1/3 provide evidence for the null hypothesis). Please note, the scale for the *Y* axis is logarithmic

Mean relative changes of the pupil size in each condition are presented in Figure 5a. In order to examine the temporal differences among the three conditions, we compared the differences between each of the two conditions over the whole time‐course of pupil measurement. Meaningful differences (${\mathrm{BF}}_{10}\geq 3\;$) are presented by the horizontal double lines.

The analysis (Figure 5) indicates that pupil size was larger for incongruent compared with both congruent and neutral conditions. The differences between incongruent and congruent trials appeared at about 570 ms after the stimulus onset and were maintained until about 1620 ms after the stimulus onset. The differences between incongruent and neutral trials appeared at about 790 ms after the stimulus onset and were maintained until about 1310 ms after the stimulus onset. However, no differences were found between congruent and neutral trials.

### 3.3 Discussion

RTs were slower in incongruent trials compared to neutral trials, which were slower than congruent trials. In contrast, pupil dilation produced a different pattern. Specifically, pupils were larger in the incongruent trials compared with both congruent and neutral trials, but no differences were found between congruent and neutral trials.

The differences in pupil size of the congruent compared with the incongruent trials indicate the existence of the information conflict. This indication for information conflict starts at about 570 ms after stimulus onset, which is a little bit earlier than the information conflict that was found for the Stroop color‐word task (Hershman & Henik, 2019). Importantly, the lack of differences between congruent and neutral trials indicates that task conflict appeared not only in the congruent trials, as in experiment 1, but also in the neutral trials that were composed of digits.

One could argue that it is not possible to distinguish temporally between the two effects due to the difference in task conflict onset. In experiment 1, we found that evidence for task conflict onset occurs before evidence for information conflict. This is in line with previous Stroop color‐word studies (e.g., Goldfarb & Henik, 2007). Moreover, there was no report of information conflict onset before the task conflict in experiment 2, due to the absence of the task conflict (difference between incongruent and neutral trials). However, the difference in the onset of task and information conflict appears to be larger in color‐word Stroop tasks than in the presented numerical Stroop task. The results might be peculiar to the numerical Stroop task or a coincidence, with the actual onset difference corresponding to the onset difference found in the word‐color Stroop task. Future studies will be needed to investigate this issue.

## 4 GENERAL DISCUSSION

Our expectations, presented at the end of the introduction were confirmed. In experiment 1, pupil measurement showed an early reverse facilitation (i.e., task conflict) followed by a late difference between incongruent and congruent trials (i.e., information conflict). RT showed information conflict with no facilitation. In experiment 2, pupil measurement showed information conflict but no reverse facilitation (i.e., no indication of task conflict). In contrast to the first experiment, in experiment 2, RT indicated both information conflict and facilitation.

In two experiments, participants were presented with two different stimuli (in most cases digits) and were asked to select the physically larger one. In experiment 1, the neutral stimuli were filled rectangles. These meaningless non‐digit stimuli did not evoke processing of the numerical dimension and as a result, did not evoke task conflict. Hence, in the same line as the color‐word version of the Stroop task, evidence for task conflict, as represented by reverse facilitation (i.e., more effort in congruent trials than in neutral trials), was observed in pupil dilation. In contrast, in experiment 2 all the conditions (including the neutrals) were composed of digits. As a result, both RTs and pupil dilation showed indications only for information conflict (i.e., more effort in incongruent trials than in congruent trials). No indication of task conflict appeared.

The current results present a dissociation between RT and pupil dilation. In experiment 1, when the neutral stimuli included no numerical information, pupil dilation showed evidence for both information and task conflicts. In contrast, RT results presented evidence for information conflict (i.e., congruent were faster than incongruent trials) but no evidence for task conflict (i.e., no differences were found between congruent and neutral trials). In experiment 2, when the neutral stimuli were numbers, pupil dilation showed evidence only for information conflict but no evidence for a task conflict. In addition, RTs were shorter in congruent than in neutral trials that were shorter than in incongruent trials. This dissociation between RT and pupil dilation has already been presented in previous Stroop pupillometry studies (Hershman et al., 2020, 2021; Hershman & Henik, 2019, 2020). Moreover, while RTs did not provide any evidence for the task conflict (i.e., no reverse facilitation), pupil dilation provided evidence for task conflict when the neutral condition did not include numerical information (experiment 1). The current work suggests that the theoretical developments (e.g., the existence of two conflicts) achieved in the Stroop color‐word task could be generalized to other tasks like the numerical Stroop task and possibly to other areas of study like numerical cognition. Together, earlier results and the current results advance our understanding regarding the component conflicts that are part of the Stroop task and possibly of other cognitive control tasks. Importantly, the understanding gained in one area regarding cognitive control and specific goal‐directed behavior could be applied in other areas. This is not a trivial achievement because we know that the correlations between various control tasks are not as high as expected from tasks supposed to indicate the same theoretical construct (i.e., control). Moreover, understanding the processes involved in goal‐directed behavior could help in understanding processes pursued in other areas, such as addictive behavior. Moeller et al. (2014) conducted the color‐word Stroop task with people presenting addictive behaviors (i.e., cocaine use disorder) and were able to show alternations in the activity in the dorsolateral prefrontal cortex and the anterior cingulate for the task error and therefore their inhibition capacity. Thus, it would be reasonable to assume that pupillometry, which in itself is connected to activity in the very same area (Murphy et al., 2014), would have some implications for the inhibition capacity as well. However, we know that addictive behavior has not one but two feature components (Ainslie, 2021). In addition to inhibition, *resolve capacities* play into the performance and must be considered. The present study does not allow any conclusions regarding this aspect of addictive behavior and future studies must be conducted to establish the connection between resolve capacities and pupillometry as well. In the context of inhibition in addictive behavior, researchers study the conflict between craving triggered by specific stimuli (e.g., “urgency to act, often without thinking, is driven by strong implicit attitude …” [Turel & Serenko, 2020, p. 2] that are in conflict with long‐term goals of the organism). We would predict that these conflicts are similar to the task conflict and not information conflict. Accordingly, addictive behavior could increase task conflict and training aimed at reducing addictive behavior could target better handling of task conflict.

## 5 SUMMARY

In the two experiments, we employed two different neutral trials. In the first experiment, the neutral trials were composed of rectangles and in the second experiment, they were composed of digits. Both conditions were designed in order to create non‐conflict trials, but they were different because we aimed to test two different conditions of non‐conflict trials. These two conditions led to different patterns of results; a reverse facilitation in the first experiment and no such reverse facilitation in the second experiment. Importantly, what seemed to be a good neutral in the numerical Stroop task (i.e., two digits) appeared not to be the best neutral if one is interested in unraveling not only information conflict but also task conflict. Put in different words, the type of non‐conflict/neutral that one uses should be tied to one's theoretical framework. If two conflicts rather than one are expected and one conflict appears to be due to tasks that may be afforded by the context or the stimulation, it is important to use the appropriate stimuli that could reveal such conflicts. This was suggested earlier for the Stroop color‐word task (Goldfarb & Henik, 2007; Hershman & Henik, 2019; Kalanthroff et al., 2018). The present study provides the first evidence for the generalizability of this idea in the numerical Stroop task.

## AUTHOR CONTRIBUTIONS

**Ronen Hershman:** Conceptualization; formal analysis; investigation; methodology; software; validation; writing – original draft. **Lisa Beckmann‏:** Conceptualization; data curation; investigation; validation; visualization; writing – original draft. **Avishai Henik:** Conceptualization; project administration; supervision; writing – review and editing.

## Footnotes

^1^
In the present study we use the term “information conflict” to indicate lexical, semantic, and response‐production‐related interference and facilitation due to the existence of two pieces of information (Banich, 2019; Zahedi et al., 2019).

^2^
The results of two participants showed no divergence between congruent and neutral trials and therefore no task conflict. In addition, the results of another participant showed no divergence between incongruent and neutral trials, indicating the absence of an information conflict. The results of these three participants were excluded from the analysis of conflict onset.
